# Predictors of Metabolic Syndrome in the Iranian Population: Waist Circumference, Body Mass Index, or Waist to Hip Ratio?

**DOI:** 10.1155/2013/198384

**Published:** 2013-03-24

**Authors:** Mojgan Gharipour, Nizal Sarrafzadegan, Masoumeh Sadeghi, Elham Andalib, Mohammad Talaie, Davood Shafie, Esmaiel Aghababaie

**Affiliations:** ^1^Isfahan Cardiovascular Research Centre, Isfahan Cardiovascular Research Institute, Isfahan University of Medical Sciences, Isfahan, Iran; ^2^Rehabilitation Research Center, Isfahan Cardiovascular Research Institute, Isfahan University of Medical Sciences, P.O. Box 81465-1148, Isfahan, Iran; ^3^Hypertension Research Centre, Isfahan Cardiovascular Research Institute, Isfahan University of Medical Sciences, Isfahan, Iran; ^4^Cardiology Department, School of Medicine, Isfahan University of Medical Sciences, Isfahan, Iran

## Abstract

This study aimed to investigate whether body mass index (BMI), waist circumference (WC), or waist to hip ratio (WHR) could be a better predictor of metabolic syndrome and, if so, what would be the cutoff points for these surrogates to appropriately differentiate metabolic syndrome in different age and sex subgroups. *Methods*. The present cross-sectional study was conducted on a sample of Isfahan Cohort Study (ICS). In total, 468 individuals (194 with and 274 subjects without metabolic syndrome) according to the National Cholesterol Education Program's Adult Treatment Panel III (ATP-III) criteria were selected. Anthropometric indices were measured and plotted using receiver-operating characteristic (ROC) curves. *Results*. According to ROC curve analysis, WC and WHR parameters were better indicators of metabolic syndrome compared to BMI in women, whereas in men WHR had a lower discriminating value compared to the other two parameters. Among these three anthropometric parameters, BMI had a lower sensitivity and WC and WHR both had a higher sensitivity for predicting metabolic syndrome in women compared with in men. The cut points for WC were nearly equal in men and women, 90.3 versus 90.0, respectively. Women had higher cut points for BMI (28.5 kg/m^2^) compared to men (26.0 kg/m^2^). Our results showed the highest sensitivity and specificity for WC cut points specially in women. To predict metabolic syndrome, we looked into optimal age-specific cut points for BMI, WC, and WHR. The results indicated that WC had the highest discriminating value compared to other indicators in the different age subgroups. The optimal cut points for all three parameters gradually increased with age. *Conclusion*. Our results demonstrated that regardless of gender and age variables, WC could be a preferred parameter for predicting metabolic syndrome compared to BMI and WHR in Iranian population.

## 1. Introduction

Metabolic syndrome is known to be a cluster of variety of cardiac and metabolic-related factors such as obesity, elevated blood pressure, glucose metabolism disturbances, and raised lipid levels leading to increased risk of mortality and morbidity [1–4]. From all these risk factors, existing diagnostic criteria emphasize the importance of body fat [5]. It has been suggested that central obesity as an indicator of body fat can be easily and cost-effectively estimated by measuring body mass index (BMI) and waist circumference (WC) which might discriminate metabolic syndrome from nonmetabolic syndrome status [6, 7].

To diagnose metabolic syndrome, different cutoff points for these anthropometric parameters have been defined to diagnose central obesity and therefore metabolic syndrome. These cutoff points are mainly derived from studies conducted on western populations [8, 9]. We believe that ethnic and racial differences in our population might require different cutoff points and or use of different anthropometric parameters for diagnosis of metabolic syndrome. There are controversies on mortality and morbidity rates related to the defined cutoff points [10] and also lack of sex and age specificity. To our knowledge, no previous studies have investigated appropriate cut points for body fat indices that could have the highest value for diagnosis of metabolic syndrome neither have demonstrated the best indicators for central obesity related to different age and sex subgroups. We therefore investigated BMI, WC, or waist to hip ratio (WHR) in Iranian population to evaluate which one could better define metabolic syndrome and what cutoff points for these surrogates are more appropriate to diagnose metabolic syndrome in different age and sex subgroups.

## 2. Methods

This is a cross-sectional study conducted on 468 participants who were selected from those enrolled in Isfahan Cohort Study which was a population-based cohort and previously reported [11, 12]. Isfahan Cohort Study (ICS) was conducted in 2001 in three central provinces in Iran including Isfahan, Arak, and Najafabad and enrolled 6504 participants with age ≥35 years old. The primary outcomes of the cohort were detecting the incidence of cardiovascular diseases (CVDs) and mortality and morbidity resulted from it in addition to the incidence of major risk factors for CVDs. All participants underwent a complete clinical examination for cardiovascular evaluation including systolic blood pressure (SBP) and diastolic blood pressures (DBP), fasting blood sugar, and fasting serum lipid indices measurements. We excluded all subjects with history of any cardiovascular or related disorders, pregnant women, breast feeding mothers, and those subjects with serious systemic illnesses from the study. The study protocol was approved by human research ethics committee of Isfahan Cardiovascular Research Institute, a WHO collaborative center. An informed consent was obtained from all participants prior to enrolment in the study. Details of ISC study were reported previously [13]. All participants were asked to fast for 8–12 hours before attending the first visit in order to obtain demographics information, a medical history, to conduct clinical examination and venous blood samples for lab tests. We first assessed coronary artery disease risk factors as per the following definitions. Current smoking history: if patients regularly smoke a tobacco product/products one or more times per day or have smoked for 30 days prior to admission [14]; hypercholesterolemia: total cholesterol ≥ 5.0 mmol/L, HDL-cholesterol ≥ 1.0 mmol/L in men, and ≥1.1 mmol/L in women, triglycerides ≥ 2.0 mmol/L [15]; hypertension: SBP ≥ 140 mmHg and/or DBP ≥ 90 mmHg and/or use of antihypertensive treatment [16]; and diabetes mellitus: symptoms of diabetes plus presence of at least one of the following lab tests including plasma glucose concentration ≥ 11.1 mmol/L, fasting plasma glucose ≥ 7.0 mmol/L, and 2-hpp ≥ 11.1 mmol/L) [17]. 

Blood pressure was measured using a standard mercury sphygmomanometer on the right arm with subjects seated and after at least 10-minute resting. Measures of WC were obtained by a measure tape of horizontal plane, midway between the inferior margin of the ribs and the superior border of the iliac crest. Body weight and height were measured by using a digital scale accurate to the nearest 0.1 kg and a wall stadiometer, respectively. To measure BMI the Quetelet formula was used (weight in kilograms divided by height in meters squared). Total cholesterol, high density lipoprotein-cholesterol (HDL-C), triglyceride, and blood glucose were assessed using a spectrophotometer. The variation coefficient was <5% for all laboratory measurements. The definition of metabolic syndrome was based on the presence of three or more components out of five defined criteria for metabolic syndrome by the amended National Cholesterol Education Program's Adult Treatment Panel III (ATP-III) [18]. The ATP-III criteria include (1) fasting triglycerides > 150 mg/dL or lipid medications; (2) SBP > 130 mmHg, DBP > 85 mmHg, or use of antihypertensive medications; (3) fasting plasma glucose > 110 mg/dL or use of diabetes medications; (4) HDL cholesterol < 40 mg/dL (men) or <50 mg/dL (women); and (5) waist circumference > 102 cm (men) or >88 cm (women). Using the previous criteria we found 194 patients who were diagnosed to have metabolic syndrome and 274 patients without metabolic syndrome.

### 2.1. Statistical Analysis

The results were presented as absolute frequencies and percentages for categorical variables and mean ± SD for continuous variables. Categorical variables were compared using chi-square test or Fisher's exact test when more than 20% of cells with expected count of less than 5 were observed. Continuous variables were also compared using *t*-test. To compare the ability of BMI, WC, and WHR to predict presence of metabolic syndrome, these indices were plotted using receiver-operating characteristic (ROC) curves. The areas under the curve (AUC) and associated 95% confidence intervals (CIs) were compared. The larger the AUC, the more accurate the test. A *P* value <0.05 was considered statistically significant. To determine the best sex- and age-related cut points for BMI, WC, and WHR in relation to the subject's metabolic syndrome we looked into the shortest distance between any point on the ROC curve and the top left corner of the *y* axis and plotted separate ROC curves for each variable and associated AUC [5]. Intercooled STATA (version 9.1) was used to analyze data and plot curves as well as to determine sensitivity and specificity. Statistical comparisons for all variables between the two genders were also performed using SPSS software (version 19.0, SPSS Inc., Chicago, IL, USA).

## 3. Results

In total, 468 subjects (194 with and 247 without metabolic syndrome) were evaluated for this study. The male to female (M/F) ratio in metabolic syndrome group was 42% to 58% and in the group without metabolic syndrome was 56% to 44% (Table 1). Percentage of current smokers was similar between men with and without metabolic syndrome. None of the females were current smokers. In both sex subgroups, the overall prevalence of high blood pressure, diabetes mellitus, and dyslipidemia was higher in the group with metabolic syndrome. The average of BMI, SBP and DBP, blood glucose, total serum cholesterol, and serum triglycerides was significantly higher in both men and women with metabolic syndrome compared to subjects without metabolic syndrome. 

**Figure 1:**
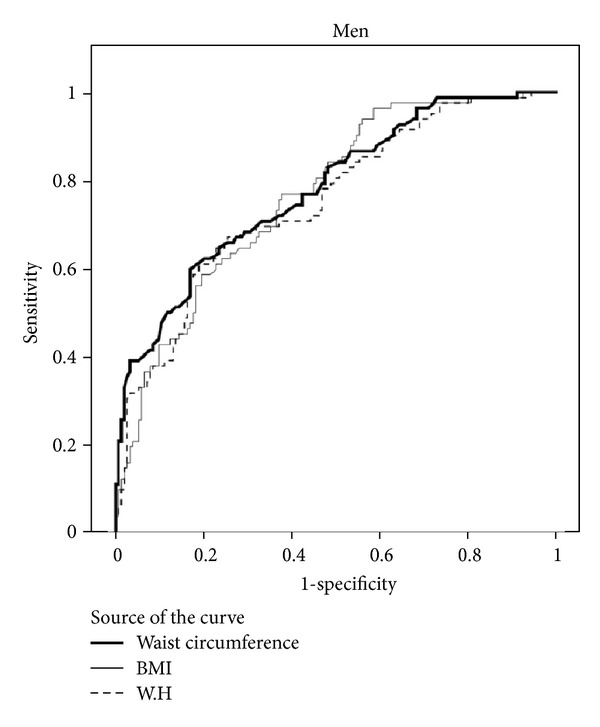


**Figure 2:**
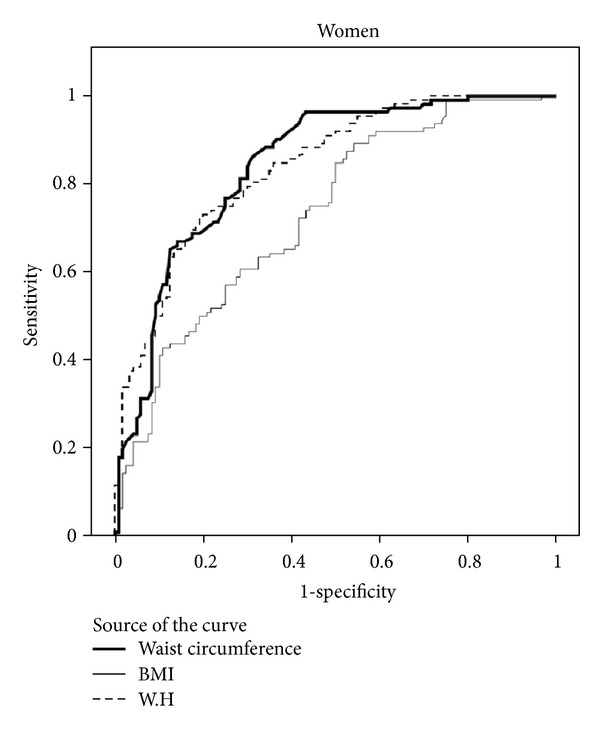


The predicting values for metabolic syndrome and corresponding AUC of BMI, WC, and WHR in both genders are shown in Table 2, Figures 1 and 2. 

We found that in women, WC (with the AUC of 0.85) and WHR parameters (with the AUC of 0.84) were better indicators of metabolic syndrome compared to BMI (with the AUC of 0.73), whereas in men WC (with the AUC of 0.78) and BMI (with the AUC of 0.77) were better indicators of metabolic syndromes and WHR (with the AUC of 0.75) had a lower discriminating value comparatively. 

In general, from the three studied anthropometric parameters, WC and WHR had a higher sensitivity and BMI had a lower sensitivity for predicting metabolic syndrome in women compared with in men. Further ROC analysis resulted in optimal gender-specific cut points for BMI, WC, and WHR together along with associated sensitivity and specificity. As shown in Table 2, the cut points for WC were nearly equal in men and women (90.3 versus 90.0), respectively. Women, on the other hand, had higher cut points for BMI (28.5 kg/m^2^) compared to men (26.0 kg/m^2^). With regard to WHR women had lower cut points compared to men. The highest sensitivity and specificity were exhibited by the WC cut points. 

The optimal age-specific cut points for BMI, WC, and WHR for predicting metabolic syndrome are presented in Table 3. Our results demonstrated that in the different age subgroups, WC had the highest AUC for discriminating metabolic syndrome compared to other indicators. All the studied anthropometric parameters had their highest sensitivity and specificity shown in the subgroup aged 50–59 years old. The optimal cut points for the three parameters gradually increased with age. 

## 4. Discussion

The aim of the present study was determining discriminating values of common anthropometric parameters for metabolic syndrome as well as discovering age- and sex-specific cutoff points for these parameters in Iranian population. According to our findings, two indices of WC and WHR were better indicators of metabolic syndrome compared to BMI in women, while WC and BMI were more preferable than for discriminating this syndrome in men [19]. On the other hand, WC had the highest value for predicting metabolic syndrome with appropriate sensitivity and specificity. The value of WC, BMI, and WHR in differentiating metabolic syndrome from nonmetabolic syndrome has been a matter of controversy. Beydoun et al. found BMI to be inferior o WC among men in general and White men in particular for prediction of metabolic syndrome, but not women [5]. What Beydoun is suggesting is consistent with other reports by Reeder et al. [20], Moreno et al. [21], and Wang et al. [22]. However, another report suggested that WC, WHR, and BMI values were equally useful indicators to identify the presence of metabolic abnormalities in Chinese population [23]. Given different results reported by variety of population-based studies in different ethnicities from western and eastern countries on the discriminating value of WC, WHR, and BMI we believe that the selection of each parameter for diagnosis of metabolic syndrome should be specified to each ethnic population. Hence, among Iranian population, WC might be the most appropriate indicator to discriminate metabolic syndrome regardless of gender and age variables. Our study also showed superiority of WC to other indices in different age subgroups. Because of correlation between WC with abdominal fat mass and because WC is more associated with cardiovascular risk compared to BMI, currently, WC and its related values are widely used as a representative indicator of abdominal adiposity [24–26]. 

In our study, the cutoff values of WC for diagnosis of metabolic syndrome were partially equal in men and women, whereas the cut points of BMI were higher for women and the WHR was higher for men. One of the main findings in our study was observation of the increasing trend of the cutoff points for all anthropometric indices with age increase. In similar studies to ours, the cutoff points of BMI in men were reported to be lower than in women, but our results showed that the cut points were considerably higher compared to what has been previously revealed in other populations [23]. In fact, due to the same reason, the currently used cut points which are derived from studies on European, American, or southeastern populations may not be applicable to Iranian ethnic groups [27]. Therefore, we recommend replacing the obtained cutoff points after validation in Iranian population. 

In the current study, we did not assess the value of the combination of anthropometric parameters in comparison with their use alone. However, some previous studies on other populations showed that WC together with BMI were good discriminating indices for metabolic syndrome [22]. Beydoun et al. indicated that instead of using just one of these parameters, it is possible to increase both sensitivity and specificity of diagnosis of metabolic syndrome by combining both WC and BMI to a practical clinical level [5]. Wrist circumference is reported as a significant predictor of diabetes in both genders of adult population. However, its predictability is independent of BMI or WC only among females. Because of its simple and easy-to-detect nature, wrist circumference could be considered as a new anthropometric assessment for prediction of type 2 diabetes and metabolic syndrome.

Other studies on Iranian population also showed that BMI and WC have the same power to predict metabolic syndrome [26–30]. Mirmiran et al. confirmed importance of waist circumference in risk stratification of metabolic syndrome in adulthood [31, 32].

## 5. Limitation

The present study is limited by its cross-sectional nature so we could not evaluate outcome measures. Consequently, the authors are mindful that differences could only be imputed from the previously documented data. 

## 6. Conclusion

Our findings demonstrated that in Iranian women, the two indices of WC and WHR were better indicators of metabolic syndrome while in men, WC and BMI were superior indicators. We observed that WC, regardless of age subgroup or gender, had the highest predicting value for discriminating metabolic syndrome with appropriate sensitivity and specificity. The optimal cut points for metabolic syndrome for all three parameters gradually increased with age.

## Figures and Tables

**Table 1:** Baseline characteristics and clinical parameters in men and women with and without MetS.

Variables		Men			Women	
	Without MetS (*N* = 154)	with MetS (*N* = 82)	*P* value	Without MetS (*N* = 120)	with MetS (*N* = 112)	*P* value
Hypertension (%)	18 (11.7)	40 (48.8)	**<0.001**	18 (15.0)	37 (33.0)	**0.001**
Diabetes mellitus (%)	4 (2.6)	29 (35.4)	**<0.001**	3 (2.5)	39 (34.8)	**<0.001**
Dyslipidemia (%)	100 (64.9)	73 (89.0)	**<0.001**	79 (65.8)	105 (93.8)	**<0.001**
Age (years)	57.58 ± 10.22	57.68 ± 9.69	0.942	54.21/8.50	56.25/9.05	0.078
BMI (kg/m^2^)	25.62 ± 3.32	29.13 ± 3.44	**<0.001**	28.41/4.61	32.26/4.53	**<0.001**
Systolic blood pressure (mm Hg)	118.47 ± 15.40	135.52 ± 16.35	**<0.001**	119.04/18.63	128.90/17.19	**<0.001**
Diastolic blood pressure (mm Hg)	77.29 ± 7.12	84.80 ± 8.05	**<0.001**	76.93/7.49	80.10/9.30	**0.005**
Fasting blood glucose (mg/dL)	83.86 ± 20.24	108.06 ± 47.16	**<0.001**	81.93/11.54	109.80/45.94	**<0.001**
Total cholesterol (mg/dL)	200.86 ± 37.52	210.85 ± 46.65	0.078	213.18/35.54	218.48/45.12	0.319
HDL cholesterol (mg/dL)	45.23 ± 11.04	38.38 ± 6.96	**<0.001**	53.80/11.56	44.79/9.31	**<0.001**
LDL cholesterol (mg/dL)	117.01 ± 25.14	117.28 ± 28.08	0.939	101.03/24.25	121.71/27.77	0.844
Triglyceride (mg/dL)	142.35/63.99	269.63/196.30	**<0.001**	134.18/60.38	232.85/123.59	**<0.001**

**Table 2:** Areas under the ROC curve cutoffs, sensitivity and specificity of WC, BMI, and WHR by sex.

	AURC (95% CI)	*P* value	Cut-off	Sensitivity	Specificity
Women					
WC	0.85 (0.79–0.90)	<0.001	90.3	86.6%	68.3%
BMI	0.73 (0.67–0.79)	<0.001	28.5	79.5%	50.8%
WHR	0.84 (0.79–0.89)	<0.001	0.88	83.0%	65.0%
Men					
WC	0.78 (0.72–0.84)	<0.001	90.0	82.9%	51.9%
BMI	0.77 (0.71–0.83)	<0.001	26.0	80.5%	54.5%
WHR	0.75 (0.69–0.82)	<0.001	0.93	80.5%	50.6%

**Table 3:** Cut points for areas under the ROC curve, sensitivity and specificity of WC, BMI, and WHR by age groups.

	AURC (95% CI)	*P* value	Cut-off	Sensitivity (95% CI)	Specificity (95% CI)
Age of 40–49 years					
WC	0.78 (0.69–0.86)	<0.001	88.5	83.3%	52.5%
BMI	0.71 (0.62–0.81)	<0.001	27.5	79.2%	50.0%
WHR	0.71 (0.63–0.80)	<0.001	0.88	81.3%	57.5%
Age of 50–59 years					
WC	0.84 (0.79–0.90)	<0.001	89.0	89.5%	62.0%
BMI	0.79 (0.73–0.85)	<0.001	27.5	81.0%	62.0%
WHR	0.79 (0.73–0.86)	<0.001	0.89	85.5%	53.7%
Age of ≥60 years					
WC	0.78 (0.70–0.85)	<0.001	92.0	81.4%	55.8%
BMI	0.77 (0.70–0.84)	<0.001	28.8	80.0%	61.6%
WHR	0.76 (0.68–0.83)	<0.001	0.93	82.9%	52.3%
